# Epidemiology, mortality and effectiveness of prophylaxis for *Pneumocystis jiroveci* pneumonia among rheumatic patients: a territory-wide study

**DOI:** 10.1186/s12941-021-00483-2

**Published:** 2021-11-11

**Authors:** Shirley Chiu Wai Chan, Ho Yin Chung, Chak Sing Lau, Philip Hei Li

**Affiliations:** Division of Rheumatology and Clinical Immunology, Department of Medicine, Queen Mary Hospital, The University of Hong Kong, Hong Kong, China

**Keywords:** Epidemiology, Prophylaxis, Rheumatology, Fungal, Pneumonia, Mortality

## Abstract

**Background:**

*Pneumocystis jiroveci* pneumonia (PJP) is an opportunistic infection affecting immunocompromised individuals. However, evidence regarding the burden and effectiveness of prophylaxis among rheumatic patients remains limited. Delineating the epidemiology and efficacy of prophylaxis among rheumatic patients is urgently needed.

**Methods:**

We performed a territory-wide cohort study of rheumatic patients in Hong Kong. All patients with a diagnosis of anti-neutrophil cytoplasmic antibody-associated vasculitis (AAV), immune-mediated myositis (IMM), rheumatoid arthritis (RA), systemic lupus erythematosus (SLE), systemic sclerosis (SSc), or spondyloarthritis (SpA) between 2015 and 2019 were included. Prevalence, frequency of prophylaxis and mortality of PJP were calculated. Number needed to treat (NNT) analysis was also performed.

**Results:**

Out of 21,587 patients (54% RA, 25% SLE, 13% SpA, 5% IMM, 2% AAV and 1% SSc), 1141 (5.3%) patients were prescribed PJP prophylaxis. 48/21,587 (0.2%) developed PJP. No patients who developed PJP received prophylaxis prior to infection. The incidence of PJP was highest among SSc, AAV, and IMM patients. Among these diseases, the majority of PJP occurred while patients were on glucocorticoids at daily prednisolone-equivalent doses of 15 mg/day (P15) or above. PJP prophylaxis was effective with NNT for SSc, AAV and IIM being 36, 48 and 114 respectively. There were 19 PJP-related mortalities and the mortality rate was 39.6%.

**Conclusion:**

PJP is an uncommon but important infection among rheumatic patients, PJP prophylaxis is effective and should be considered in patients with SSc, AAV and IMM, especially those receiving glucocorticoid doses above P15.

## Introduction

*Pneumocystis jiroveci* pneumonia (PJP) is an opportunistic infection affecting immunocompromised individuals. Historically, PJP was the most common cause of death among patients with human immunodeficiency virus (HIV) but its incidence has declined over the past decades following the introduction of highly active antiretroviral therapy and routine PJP prophylaxis [1, 2]. In contrast, PJP has become an increasingly important cause of atypical pneumonia among non-HIV immunocompromised patients due to the expanding armamentarium in immunosuppressive and chemotherapeutic therapies [3]. PJP in non-HIV patients runs an aggressive course and is associated with even higher mortality compared with HIV patients [4]. Due to its high mortality, PJP prophylaxis is commonly prescribed in many immunocompromising conditions and various guidelines have been established in oncology, bone marrow and solid organ transplant (SOT) [5–7]. Co-trimoxazole and aerolised pentamidine (especially for glucose-6-phosphate dehydrogenase deficient patients) are the preferred first-line agents for PJP prophylaxis in Hong Kong [8].

Patients with rheumatic diseases are at risk of PJP due to their immunocompromised state contributed by both the diseases and the use of immunosuppressive therapies. The risk of PJP varies across different rheumatic diseases. For example, it has been shown that granulomatosis with polyangiitis is associated with highest risk, while rheumatoid arthritis (RA) is associated with the lowest risk of PJP [9]. Use of immunosuppressive therapies, including glucocorticoids (GC) especially at high dose over a prolonged period of time and cyclophosphamide (CYC) have also been reported as risk factors for developing PJP [10]. Although some studies have demonstrated the efficacy and safety of PJP prophylaxis, these have been limited to only selected rheumatic diseases [11, 12]. In comparison to other specialties, international consensus and guidelines are still lacking for rheumatic patients, largely due to absence of comprehensive observational studies to guide recommendations.

In order to address these shortcomings, we took advantage of Hong Kong’s comprehensive electronic health record system and reviewed all rheumatic patient records of our territory-wide database. The Hospital Authority (HA) of Hong Kong has one of world’s largest clinical information systems with a unified health record database for more than 7.1 million unique patients. Using this population-wide data, we performed a comprehensive study to delineate the incidence, mortality and efficacy of PJP prophylaxis in rheumatic patients of Hong Kong.

## Methods

This was an observational, longitudinal cohort study based on data retrieved from the territory-wide electronic healthcare database (Clinical Data Analysis and Reporting System) of HA. The primary endpoint was the incidence of PJP in rheumatic patients. The secondary endpoint was the effectiveness of PJP prophylaxis. HA is the sole public-funded healthcare provider of Hong Kong and serves a population of over 7 million patients through 43 hospitals, 49 specialist out-patient clinics and 73 general out-patient clinics; covering around 90% of all secondary and tertiary care of Hong Kong [12]. All data from this database is anonymized (having only identity numbers without patient contact details) to protect patient identity and has been extensively used for conducting other high-quality large big-data studies [13–16].

All patients with a diagnosis of anti-neutrophil cytoplasmic antibody-associated vasculitis (AAV), immune-mediated myositis (IMM), rheumatoid arthritis (RA), systemic lupus erythematosus (SLE), systemic sclerosis (SSc), or spondyloarthritis (SpA) were included. All relevant longitudinal follow-up data from 1st January 2015 to 31st December 2019 was retrieved. Rheumatic diagnoses were defined based on diagnosis code using the International Classification of Diseases, Ninth Revision (ICD-9). Other essential clinical information including patients’ demographics, medication prescriptions, blood tests and mortality (and cause, if any) were recorded. Medication prescriptions including the use of immunosuppressive agents within one year prior to PJP were analysed for patients with PJP. Blood tests included complete blood counts with differentials; liver and renal function tests at the time of PJP were evaluated.

PJP was defined as one of the following: ICD-9 diagnosis code and/or positive microbiological results from deep respiratory tract specimens including direct microbiological identification and/or detection by in-house quantitative polymerase chain reaction (PCR). Direct microbiological identification referred to the visualization of PJP cysts using methenamine silver stain. A positive PCR in the absence of clinical manifestations was not considered as PJP, and a PCR cut-off value was not used. PJP prophylaxis was defined as prescription of a prophylactic dose of co-trimoxazole for at least 2 weeks and/or aerolised pentamidine. This study was approved by the Institutional Review Board of the University of Hong Kong/Hospital Authority Hong Kong West Cluster.

### Statistical analysis

Baseline characteristics of patients were compared using Student’s t test for continuous and the χ^2^ test between patients with and without PJP. Prevalence of PJP, prophylaxis and mortality were calculated. Number needed to treat (NNT) analysis was performed based on absolute risk reduction of PJP in patients with and without prior PJP prophylaxis.

## Results

A total of 21,587 unique rheumatic patients were analysed. The mean age was 58.1 ± 17.4 years and the male: female ratio was 1:2.3 (Table 1). Majority of patients had RA (54%), followed by SLE (25%), SpA (13%), IMM (5%), AAV (2%) and SSc (1%).

**Table 1:** Baseline characteristics, frequency of PJP and prophylaxis prescription

	Total	PJP	No PJP	P value	Prophylaxis	No prophylaxis	P value
All patients	21,587	48 (0.2%)	21,539 (99.8%)		1141 (5.3%)	20,446 (94.7%)	
Age	58.1 ± 17.4	58.5 ± 18.4	58.1 ± 17.4	0.85	52.8 ± 17.7	58.3 ± 17.3	< 0.01
Male	5822 (27.0%)	16 (33.3%)	5806 (27.0%)	0.32	285 (25.0%)	5537 (27.1%)	0.12
Prophylaxis	1141 (5.3%)	0	1141 (5.3%)	0.10	–	–	–
RA	11,646	13 (0.1%)	11,633 (99.9%)		72 (0.6%)	11,574 (99.4%)	
Age	63.4 ± 15.8	68.3 ± 17.3	13.4 ± 15.8	0.27	63.7 ± 14.1	63.4 ± 15.8	0.89
Male	2507 (21.5%)	5 (38.5%)	2502 (21.5%)	0.14	17 (23.6%)	2490 (21.5%)	0.67
Prophylaxis	72 (0.6%)	0	72 (0.6%)	0.78	–	–	–
SLE	5460	22 (0.4%)	5438 (99.6%)		629 (11.5%)	4831 (88.5%)	
Age	48.8 ± 16.3	46.8 ± 14.2	48.8 ± 16.4	0.57	46.4 ± 16.6	49.1 ± 16.3	< 0.01
Male	551 (10.1%)	7 (31.8%)	544 (10.0%)	< 0.01	73 (116%)	478 (9.9%)	0.18
Prophylaxis	629 (11.5%)	0	629 (11.6%)	0.09	–	–	–
SpA	2918	0	2918 (100.0%)		49 (1.7%)	2869 (98.3%)	
Age	51.8 ± 16.3	–	51.8 ± 16.3	–	60.0 ± 14.6	51.7 ± 16.3	< 0.01
Male	2229 (76.4%)	–	2229 (76.4%)	–	42 (85.7%)	2187 (76.2%)	0.12
Prophylaxis	49 (1.7%)	–	49 (1.7%)	–	–	–	–
IMM	1026	7 (0.7%)	1019 (99.3%)		220 (21.4%)	806 (78.6%)	
Age	61.8 ± 15.9	66.6 ± 14.6	61.8 ± 15.9	0.43	59.7 ± 13.3	62.4 ± 16.5	0.01
Male	343 (33.4%)	2 (28.6%)	341 (33.5%)	0.78	78 (35.5%)	265 (32.9%)	0.47
Prophylaxis	220 (21.4%)	0	220 (21.6%)	0.17	–	–	–
AAV	430	6 (1.4%)	424 (98.6%)		135 (31.4%)	295 (68.6%)	
Age	65.0 ± 17.8	64.0 ± 18.0	65.0 ± 17.8	0.89	63.7 ± 18.4	65.6 ± 17.5	0.30
Male	177 (41.2%)	2 (33.3%)	175 (41.3%)	0.70	67 (49.6%)	110 (37.3%)	0.02
Prophylaxis	135 (31.4%)	0	135 (31.8%)	0.10	–	–	–
SSc	109	2 (1.8%)	107 (98.2%)		36 (33.0%)	73 (67.0%)	
Age	54.4 ± 15.9	79.0 ± 14.1	54.0 ± 15.6	0.03	52.5 ± 18.5	55.4 ± 14.4	0.37
Male	15 (13.8%)	0	15 (14.0%)	0.57	8 (22.2%)	7 (9.6%)	0.07
Prophylaxis	36 (33.0%)	0	36 (33.6%)	0.32			

*PJP Pneumocystis jiroveci* pneumonia; *SSC* systemic sclerosis, *AAV* ANCA-associated vasculitis; *IMM* immune-mediated myositis; *SLE* systemic lupus erythematosus; *RA* rheumatoid arthritis; *SpA* spondyloarthritis

Between 2015 and 2019, 1141 (5.3%) patients were prescribed PJP prophylaxis and 48 (0.2%) developed PJP. The majority 1109 (97.2%) of patients were prescribed oral co-trimoxazole and 32 (2.8%) were prescribed aerolised pentamidine. No patients who developed PJP were prescribed prophylaxis prior to infection. There was no significant association between age and PJP. Among SLE patients, male sex was associated with PJP. PJP prophylaxis was prescribed more frequently among younger patients, especially in IMM and SLE, but not in SpA (Table 1).

**Fig. 1:**
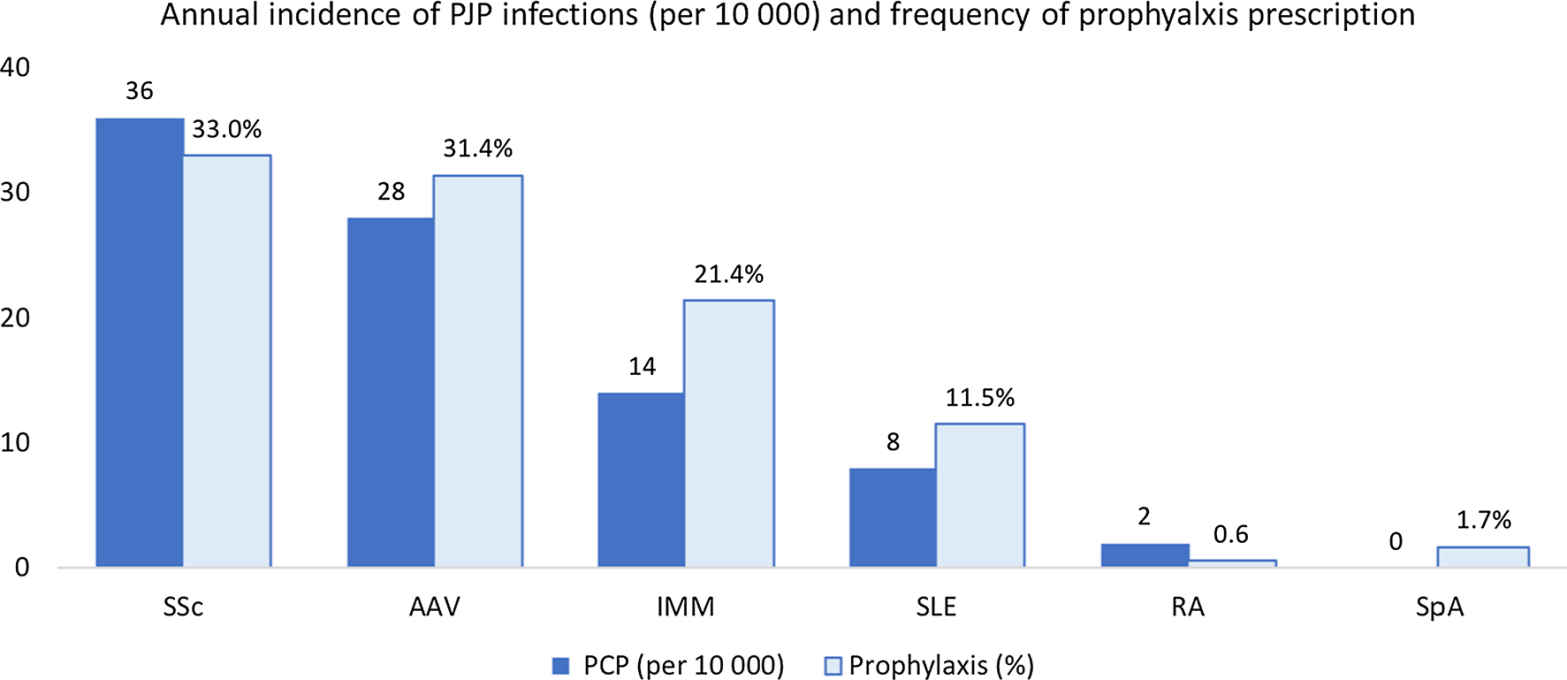
Annual incidence of PJP and prophylaxis prescriptions among different rheumatic disease categories

Among different rheumatic diseases, the risk of PJP was highest among patients with SSc, AAV, and IMM with annual incidence rates of PJP 3.6, 2.8 and 1.4 per 1,00,000 patient-year respectively. The frequency of PJP prophylaxis prescription followed a similar pattern with the highest prescription rates for patients with SSc (33.0%), AAV (31.4%) and IIM (21.4%), followed by SLE (11.5%) and RA (0.6%) (Fig. 1).

**Fig. 2:**
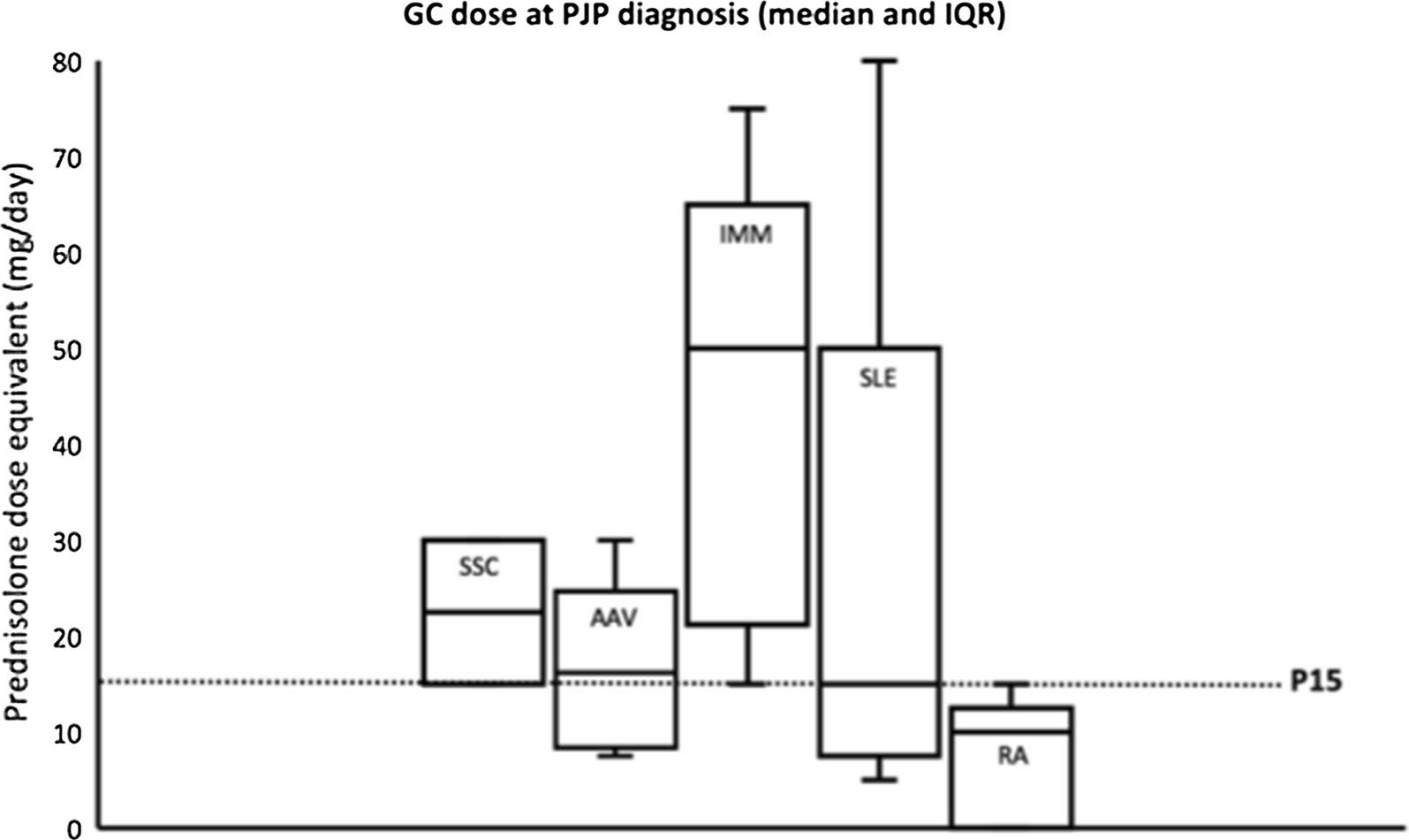
Box-and-whisker plot of GC dose (median and interquartile range) at PJP diagnosis. *GC* glucocorticoid; *PJP Pneumocystis jiroveci* pneumonia; *IQR* interquartile range; *P15* glucocorticoid at 15 mg daily prednisolone-equivalent dose; *SSC* systemic sclerosis; *AAV* ANCA-associated vasculitis; *IMM* immune-mediated myositis; *SLE* systemic lupus erythematosus; *RA* rheumatoid arthritis

Absolute risk reduction of PJP was calculated for each disease categories in patients with and without PJP prophylaxis. The NNT to prevent one PJP were lowest among patients with SSc (36), AAV (48) and IIM (114) (Table 2). Among the disease categories with highest PJP risk, the majority of PJP occurred at P15 or above (100% in SSc and IIM, 66.7% in AAV) (Fig. 2). Out of the 48 patients with PJP, there were 19 PJP-related mortalities. The mortality rate of PJP among rheumatic patients in this study was 39.6%.

**Table 2:** Effectiveness of PJP prophylaxis among different rheumatic diseases

Diagnosis	Total	Ever PJP	No PJP	ARR (%)	NNT
All patients	21,587	48	21,540		435
Prophylaxis		0	1142	0.23	
No prophylaxis		48	20,398		
RA	11,646	13	11,633		909
Prophylaxis		0	72	0.11	
No prophylaxis		13	11,561		
SpA	2918	0	2918	–	–
Prophylaxis		0	49		
No prophylaxis		0	2869		
SLE	5460	22	5438		217
Prophylaxis		0	629	0.46	
No prophylaxis		22	4809		
IMM	1026	7	1019		114
Prophylaxis		0	220	0.88	
No prophylaxis		7	799		
AAV	430	6	424		48
Prophylaxis		0	135	2.08	
No prophylaxis		6	289		
SSc	109	2	107		36
Prophylaxis		0	36	2.81	
No prophylaxis		2	71		

*PJP Pneumocystis jiroveci* pneumonia; *SSC* systemic sclerosis; *AAV* ANCA-associated vasculitis; *IMM* immune-mediated myositis; *SLE* systemic lupus erythematosus; *RA* rheumatoid arthritis; *SpA* spondyloarthritis; *ARR* absolute risk reduction; *NNT* number needed to treat

## Discussion

Our longitudinal study of over 21,000 rheumatic patients over a 5-year period is the largest epidemiological study of PJP and PJP prophylaxis ever reported. Our comprehensive territory-wide database also allowed us to uniquely calculate the estimated annual incidence of PJP among rheumatic patients from the entire region of Hong Kong. We confirm that the risk of PJP was highest among patients with SSc, AAV and IMM; as well as to identify a “threshold” GC dose of  >  P15 of which the majority of PJP occurred.

In contrast to other immunocompromising conditions, the risk of PJP is relatively low for rheumatic patients. Even among patients with SSc (highest risk), the annual incidence rate was only 3.6 per 1,00,000 patient-year, in comparison to up to 430 per 1,00,000 patient-year in SOT patients, and 7 per 1,00,000 patient-year in HIV patients [17, 18]. Despite a seemingly lower risk, PJP still poses a significant health burden with a mortality rate of around 40%—which is higher than previous reports of PJP-related mortality of 33.3% among HIV patients in Hong Kong [19].

Our unique and comprehensive study shows that the overall risk of PJP in general rheumatic patients is low, especially compared to other immunocompromising conditions. Higher incidence rates have been previously reported in smaller studies, but those studies were mostly limited to only including hospitalized patients [20]. In contrast, our data more accurately reflects the burden of PJP across entire rheumatic populations, including both community-based and hospitalized patients. A stratified approach is important taking account of patients’ factors, disease categories and immunosuppressants use to identify rheumatic patients at risk of PJP to guide the prescription PJP prophylaxis. For example, the Joint European League against Rheumatism and European Renal Association–European Dialysis and Transplant Association guideline recommended patients with AAV to receive PJP prophylaxis when treated with cyclophosphamide [21]. However, guidelines for other rheumatic diseases are currently lacking and epidemiology data, such as from this present study, would be imperative to inform these decisions.

Our study identified a “threshold” GC dose of which the majority of PJP occurred. Several mechanisms have been proposed to explain the predisposition to PJP in patients on GC. Firstly, GC reduces the numbers of circulating lymphocytes by enhancing apoptosis, including CD4  +  T cells which are essential in coordinating anti-*Pneumocystis* immunity [22]. Alveolar macrophages are the primary resident phagocytes responsible for the clearance of pneumocystis from the lung. Under the action of GC, key transcription factors such as mitogen-activated protein kinase and nuclear factor-κB are also inhibited, leading to downregulation of pro-inflammatory cytokines secretion by alveolar macrophages [22]. In our study, most cases of PJP in the highest risk disease groups (SSc, AAV and IMM) occurred when the patients were receiving  ≥  P15, which was in line with the findings from previous studies. Although the CD4 cell count is a good marker to guide PJP prophylaxis among HIV patients, studies examining its role in non-HIV patients remain limited [8]. Further studies are needed to evaluate whether absolute lymphocyte and CD4 counts (independent of GC effect) will be useful to predict the risk of PJP in rheumatic patients.

PJP prophylaxis demonstrated extreme effectiveness in this present study, with zero cases of PJP among the 1141 (5.3%) patients who were prescribed either oral co-trimoxazole and inhaled pentamidine. Despite its tremendous effectiveness, the potential adverse effects and risks of PJP prophylaxis must also be taken into consideration. The potential side effects of co-trimoxazole can range widely from mild to severe skin rashes (including life-threatening reactions such as severe cutaneous adverse drug reactions), leukopenia, liver and renal dysfunction [23]. Aerolised pentamidine can cause acute respiratory reactions during inhalation [23]. According to a Korean study by Park et al., the number needed to harm was 131 for co-trimoxazole [11]. Based on the NNT calculations, we recommend that PJP prophylaxis should be initiated for SSc, AAV and IIM especially when prescribed a GC doses above P15.

Limitations of this study stem from its observational nature. Even though the prescription patterns for PJP prophylaxis correlated with the frequency of PJP in different rheumatic disease diseases, prophylaxis was prescribed according to individual physician discretion and the criteria for prophylaxis were not standardized. We were unable to investigate or control for other potential factors which may have influenced prescription decisions (such as centre-specific or physician-specific preferences). Dedicated prospective studies investigating the optimal strategies and cost-utility of PJP prophylaxis are underway. Furthermore, despite being a very comprehensive study of all patients in the public sector, a minority (< 10% in Hong Kong) of patients might receive medical care solely from private physicians and have been missed in the current study. Additionally, although this study evaluated the risk–benefit ratio of PJP prophylaxis across different rheumatic disease categories, further studies for cost-effectiveness would be useful. However, the absolute cost of oral cotrimoxazole at standard prophylaxis dose in Hong Kong costs less than USD 1 dollar per week and adverse drug reactions remain uncommon. The cost of prophylaxis would unlikely contribute to significant cost burden, especially in comparison to the cost of healthcare utilization and productivity loss associated with PJP. However, further local studies on potential harm of PJP prophylaxis and number needed to treat analysis (especially on the use of aerolised pentamidine in the era of COVID) are urgently needed.

## Conclusion

In conclusion, PJP is an uncommon but important infection among rheumatic patients with high mortality. PJP prophylaxis is effective and should be considered in patients with SSc, AAV and IMM, especially in those receiving glucocorticoid doses above P15.

## Data Availability

The datasets used and/or analyzed during the current study are available from the corresponding author on reasonable request.

## Abbreviations

AAV: Anti-neutrophil cytoplasmic antibody-associated vasculitis
CYC: Cyclophosphamide
GC: Glucocorticoids
HA: Hospital authority
HIV: Human immunodeficiency virus
ICD-9: International Classification of Diseases, Ninth Revision
IMM: Immune-mediated myositis
NNT: Number needed to treat
OR: Odds ratio
P: Prednisolone
PJP: *Pneumocystis* *jiroveci* pneumonia
RA: Rheumatoid arthritis
SLE: Systemic lupus erythematosus
SOT: Solid organ transplant
SpA: Spondyloarthritis
SSc: Systemic sclerosis
